# A Comparison of Success Rates of Embryo Transfer on Weekdays and Weekends 

**DOI:** 10.22074/ijfs.2016.4768

**Published:** 2016-04-05

**Authors:** Pinar Solmaz Hasdemir, Melek Bulut Kamali, Esat Calik, Hasan Tayfun Ozcakir

**Affiliations:** 1 Celal Bayar University Medical School, Department of Obstetrics and Gynecology, Manisa, Turkey; 2 Celal Bayar University Infertility Research and Treatment Center, Grand Medical Hospital IVF Center, Manisa, Turkey

**Keywords:** Embryo Transfer, *In Vitro* Fertilization, Pregnancy

## Abstract

**Background:**

The aim of this study is to examine the effect of the embryo transfer (ET) day on clinical pregnancy success rates in *in vitro* fertilization-ET (IVF-ET) cycles.

**Materials and Methods:**

In this retrospective study, we divided patients with infertility who underwent IVF-ET with fresh embryos into two groups depending on whether the ET was performed on weekdays or weekends. The main outcome measure was to compare the clinical pregnancy rates of patients with similar demographic and clinical characteristics who underwent ET on weekdays or weekends.

**Results:**

A total of 188 patients underwent IVF-ET on weekdays (n=156) or weekends (n=32). Both groups had similar demographic and cycle characteristics. The overall pregnancy rate was 42.8%. Among the study groups, the weekday group had a 40.2% ET success rate and the weekend group had a 54.8% success rate (P=0.517). Although no statistically significant difference existed between the two groups, we observed an absolute 14.6% increase in pregnancy rate for ETs performed during weekends compared to those performed on weekdays, with a 35% statistical power.

**Conclusion:**

ETs performed during weekends were more successful than ETs performed during weekdays with an absolute 14.6% increase in clinical pregnancy rate. This finding should be confirmed by conducting further studies with larger groups of patients.

## Introduction

Infertility is a common health care problem with a prevalence of 4 to 14% worldwide (1). Improvements in the success rates of the *in vitro* fertilization-embryo transfer (IVF-ET) cycles can benefit couples from the physical, psychiological and financial points of view. Thus, every possible factor which can potentially improve the success of an ET should be considered. 

IVF-ET cycles were started at the beginning of the natural cycle in the first years of assisted reproductive technologies. Conventional ovarian hyperstimulation was later replaced by controlled ovarian stimulation to avoid the need for clinical and laboratory staff to be on duty during weekends (2). Since then, no studies have compared pregnancy success rates between weekdays and weekends. To the best of our knowledge, this is the first study that compares the effect of the ET day on clinical pregnancy success rates in IVF-ET cycles. 

## Materials and Methods

This was a retrospective observational study based on a review of medical records from Celal Bayar University Infertility Research and *In vitro* Fertilization Center. We included patients who referred to our clinic because of primary or secondary infertility and underwent assisted reproduction (IVF-ET) with a gonadotropin-releasing hormone (GnRH) antagonist protocol between August 2013 and January 2015. 

A total of 188 patients were divided into two groups, depending on whether the ET performed on weekdays (n=156) or weekends (n=32). Clinical characteristics that included age, duration of infertility, and body-mass index (BMI) were collected for each patient. The results of sperm analysis were collected from each patient. We defined abnormal sperm analysis as any abnormality in one of the following parameters: total count, morphology and/ or motility. Serum follicle-stimulating hormone (FSH), estradiol (E_2_) and luteinizing hormone (LH) levels were measured at the beginning of the ovulation induction and at the day of human chorionic gonadotropin (hCG) administration (E_2_ and LH). Ovulation induction protocol, oocyte retrieval days, ET days, endometrial thickness at the beginning of ovulation induction and day of oocyte retrieval, and clinical pregnancy rates were collected for each patient. Only first ovulation induction cycle of each patient was included in the study.

Exclusion criteria for IVF-ET were high serum FSH (>13 mIU/mL), serum LH (>13 mIU/mL) or serum E_2_ (>80 pg/mL) levels, use of long or short ovulation induction protocols, patients with unavailable or arrested embryos, chemical pregnancies and pregnancies with gestational sac without fetal heart beat. Also excluded were patients with IVF-ET cycles with cryopreserved embryos and those with a history of >4 IVF cycle failures. 

## Ovulation induction protocol

We used the antagonist protocol in each patient with the same ovulation induction agents. Ovulation induction was started with recombinant FSH (r-FSH, Puregon®, Australia) or human menopausal gonadotropin (HMG, Menogon®, Switzerland). GnRH antagonist (Orgalutran®, Australia, 0.25 mg/day) was added to the cycle on 5^th^ to 7^th^ day depending on the clinical characteristics of the patient. Ovulation was triggered by intramuscular hCG (Pregnyl®, Germany, 10000 IU) when at least two follicles larger than 17 mm in diameter were detected on ultrasound. Oocyte retrieval was performed 36 hours after the injection by the same operator. ETs were performed between the 2nd and 5th days after oocyte retrieval depending on the embryo characteristics; all transfers were performed with the same technique, equipment, and by the same operator. One embryo was transferred in one cycle in patients younger than 35 years of age and two embryos (in case of available two good quality embryos) in patients equal or older than 35 years of age based on legal arrangements in Turkey. Patients received luteal phase support by micronized vaginal progesterone (Progestan, 800 mg/day) from the day of oocyte retrieval which continued until a pregnancy test was performed.

## Main outcome measure

This study compared the pregnancy rates of patients with similar clinical and cycle characteristics who underwent ET on weekdays versus weekends. We considered the primary outcome measure to be the clinical pregnancy rate. A blood pregnancy test was performed 9 to 12 days after ET and ultrasound was performed 2 weeks later if the pregnancy test was positive. Clinical pregnancy was defined as presence of a gestational sac with a fetal heart beat. 

## Ethical consent

The research protocol was approved by the Institutional Review Board of Celal Bayar University Medical School (no. 20478486-302) on August 27, 2014. 

## Statistical analysis

Statistical analysis was performed with IBM SPSS Statistics 15.0 (SPSS Inc., USA). The Shapiro-Wilk test was used to calculate whether the numeric variables had normal distribution. For normally distributed variables, we used the student’s t test to analyze any differences in the distributions of the patients’ characteristics. The Mann-Whitney U test assessed abnormally dis50 tributed variables. Cross-tables and chi square analysis were employed in the evaluation of the categorical data. P value<0.05 was considered statistically significant. A power analysis was made and the effect size measured to determine the timing (weekdays versus weekends) of ET on the pregnancy success rate. 

## Results

Overall, there was a 42.8% pregnancy rate. The rate of clinical pregnancy was 40.2% in the weekdays group and 54.8% in the weekend group (P=0.517) with a power of 35% and an effect size of 15%. Although there was no statistically significant difference between the two groups, an absolute 14.6% increase in success rate existed for ETs performed during weekends compared to those performed on weekdays. 

Table 1 summarizes the baseline and cycle characteristics of the study population. Abnormal sperm analysis results existed in 69.5% of those in the ET weekdays group versus 72.4% of those in the ET weekend group (P=0.755). Both groups had a history of 0-5 intra-uterine insemination (IUI) treatments and/or 0-3 IVF programs. The percentage of IUI treatments and/or IVF programs were similar in both groups, with 54.4% of patients who underwent IUI in the weekdays group compared to 43.8% in the weekend group (P=0.442) and 29.9% who under went IVF in the weekdays group compared 31.2% who underwent IVF in the weekend group (P=0.885). The type of transferred embryos were cleaved-staged embryos in 83.9% and blastocysts in 16.1% in the weekdays group and cleaved-staged embryos in 87.5% and blastocysts in 12.5% in the weekend group. A single embryo was transferred in 78.2% of weekday patients and in 93.7% of the weekends group. Two embryos were transferred in 21.8% of weekday patients and in 6.3% of patients in the weekends group. 

**Table 1:** Baseline and cycle characteristics of the patients that underwent embryo transfer on weekdays and weekends


	ET weekdays n=156	ET weekends n=32	P value

Age of women (Y)	31.17 ± 4.34*	29.87 ± 3.91*	0.119
Age of man (Y)	33.90 ± 4.39*	32.48 ± 4.19*	0.125
Duration of infertility (Y)	5.50 (1-20)**	6 (1-22)**	0.963
BMI	25.72 ± 4.41*	24.31 ± 3.79*	0.095
Baseline FSH level (mIU/mL)	7.36 ± 3.19*	7.79 ± 2.19*	0.565
Baseline E_2_ level (pg/mL)	51.57 ± 33.61*	46.89 ± 22.06*	0.555
Baseline LH level (mIU/mL)	5.15 ± 2.83*	4.99 ± 1.92*	0.805
Baseline endometrial thickness (mm)	5.38 ± 2.51*	5.58 ± 2.06*	0.737
Dosage of induction agent (IU/day)	187.50 (100-300)**	175 (100-300)**	0.150
Retrieved follicle (n)	7.40 ± 4.75*	7 ± 3.38*	0.723
M2 oocytes (n)	6.05 ± 3.68*	5.26 ± 2.66*	0.376
Grade 1 embryos (n)	2.9 ± 3.38*	3 ± 2.69*	0.909
Fertilization rate	4.18 ± 2.61*	3.84 ± 1.77*	0.590
E_2_ level at hCG day (pg/mL)	1335.75 ± 910.33*	3.84 ± 1.77*	0.935
LH level at hCG day (mIU/mL)	3.22 ± 3.11*	6.64 ± 9.44*	0.146
Endometrial thickness (mm)	9.87 ± 2.56*	9.44 ± 2.61*	0.388
Mean transferred embryo (n)	1.29 ± 0.45*	1.15 ± 0.37*	0.174
ET day after OPU	3 (2-6)**	3 (2-5)**	0.344


*; Mean ± SD, **; Median (minimum-maximum), BMI; Body-mass index, M2; Metaphase II, ET; Embryo transfer, OPU; Ovum pick-up, P<0.05 was considered to be statistically significant, FSH; Follicle-stimulating hormone, LH; Luteinizing hormone, E_2_; Estradiol and hCG; Human chorionic gonadotropin.

### Discussion

IVF involves a complex series of steps, including controlled ovarian hyperstimulation, oocyte retrieval, fertilization, embryo culture, and uterine transfer. Many studies have described factors that influence the success rate of implantation and clinical pregnancy rates in IVF-ET cycles (3-16), including endometrial receptivity (4-6), treatments targeted to improve implantation (5, 7, 8), the ET technique (9, 10), transfer day of the conception material (11, 12), serum E_2_ level during the IVF cycle (4th day) (13), decrease in serum E_2_ level after hCG administration (14), the relationship between E_2_ and LH levels (15),and type of the agentused in ovulation induction (16). 

Craig et al. (17) recently showed that acupuncture on the day of ET had a detrimental effect on clinical pregnancy rates, although a Cochrane analysis did not confirm this finding (18). Manheimer et al. (19) have reported a small positive effect of acupuncture on ET rates. Acupuncture is known to increased nitric oxide (NO) production and vasodilatator effect via relaxation of the smooth muscles of the vessels (20). It is clear from the current literature that many factors which affect the success of IVF-ET procedures remain to be clarified. 

Uterine receptivity and implantation is one of the most enigmatic parts of the fertilization process. The junctional zone (JZ), or subendometrial layer of the myometrium, is the most popular part of the uterus related to this subject. The degree of JZ contractility has been shown to change through the ovarian cycle. Increased contractility just before ET significantly reduces the success rate of ET (12,21). It is known that uterine manipulation during ET has a negative effect on pregnancy rates and is possibly related to increased uterine JZ contractility with mechanical effect (10,22). JZ contractility may be regulated by local synthesis of NO within the uterus and high progesterone levels. It is believed to be related to uterine receptivity although the mechanism is unclear (23). A higher implantation success rate with frozen-thawed ETs has been shown in recent studies. This is thought to be related to the potential detrimental effect of endometrial receptivity induced by the high hormonal environment in fresh ET cycles (5,24). 

We believe that every possible factor which can potentially improve the success of ET should be considered. To the best of our knowledge, there is no data that compares the success rate of ET performed on weekdays versus weekends. We have found an absolute 14.6% increase in success rate for ETs performed during weekends compared to those performed on weekdays. There is no known possible factor for increasing the implantation rate during weekends. The factors that underline the difference in the current study should be investigated. This difference may be due to patient and/ or IVF team related factors. Saturdays are more relaxing days in terms of crowdedness and work load. This may help to perform easier ETs with less JZ contractions. A relaxing clinic environment as well as patient mood during the weekends may decrease patient anxiety and positively effect hormonal status related to the implantation process. 

There are several limitations in this study. Miscarriage after fetal heart beats and live birth rates were not determined. The number of patients in the study population was relatively low. According to the statistical power analysis, a 35% power was reached in this study. In order to reach a statistical significance with a 14.6% difference between the two groups, 485 patients in the weekdays group and 97 patients in weekend group were required. A nearly threefold higher patient number would be needed to reach statistical significance. In order to reach this number of patients, approximately a 5-year study would be needed at our center. Therefore, the current study findings should be confirmed with studies powered with larger numbers of patients. 

## Conclusion

ETs performed during the weekends compared to weekdays can be more successful. We strongly believe that large prospective studies are required to demonstrate whether ETs performed during the weekends are more successful in terms of clinical pregnancy rates compared to those performed on weekdays and, if so, to identify the potential reasons that improve the success rate of IVF-ET cycles. 
